# Supplementary material for: Pyrazole-Based Water-Soluble Dendrimer Nanoparticles as a Potential New Agent against Staphylococci
Source: Biomedicines. 2021 Dec 23;10(1):17. doi: 10.3390/biomedicines10010017 (PMC8773120; doi:10.3390/biomedicines10010017)
Supplement: Supplementary file 1 [file biomedicines-10-00017-s001.zip › biomedicines-1505975-supplementary.pdf]

Supplementary Materials

Pyrazole-Based Water-Soluble Dendrimer Nanoparticles as A Potential New Agent Against Staphylococci

Silvana Alfei [1,*], Chiara Brullo [1], Debora Caviglia [2], Gabriella Piatti [2], Alessia Zorzoli [3], Danilo Marimpietri [3], Guendalina Zuccari [1], and Anna Maria Schito [2]

[1] Department of Pharmacy (DIFAR), University of Genoa, Viale Cembrano, 16148 Genoa, Italy; brullo@difar.unige.it (C.B.), zuccari@difar.unige.it (G.Z.)
[2] Department of Surgical Sciences and Integrated Diagnostics (DISC), University of Genoa, Viale Benedetto XV, 6, 16132 Genoa, Italy; amschito@unige.it (A.M.S.), gabriella.piatti@unige.it (G.P.), Cavigliad86@gmail.com (D.C)
[3] Stem Cell Laboratory and Cell Therapy Center, IRCCS Istituto Giannina Gaslini, via Gerolamo Gaslini 5, 16147 Genoa Italy; alessiazorzoli@gaslini.org (A.Z.), danilomarimpietri@gaslini.org (D.M.)
* Correspondence: alfei@difar.unige.it; Tel.: +39 010 355 2296 (S.A.)

Section S1. Synthesis and Characterization of BBB4-loaded Dendrimer Nanoparticles (BBB4-G4K NPs) [16].

S1.1. Preparation of BBB4-Loaded Dendrimer NPs (BBB4-G4K NPs)

The BBB4-G4K NPs were prepared according to Scheme S1.

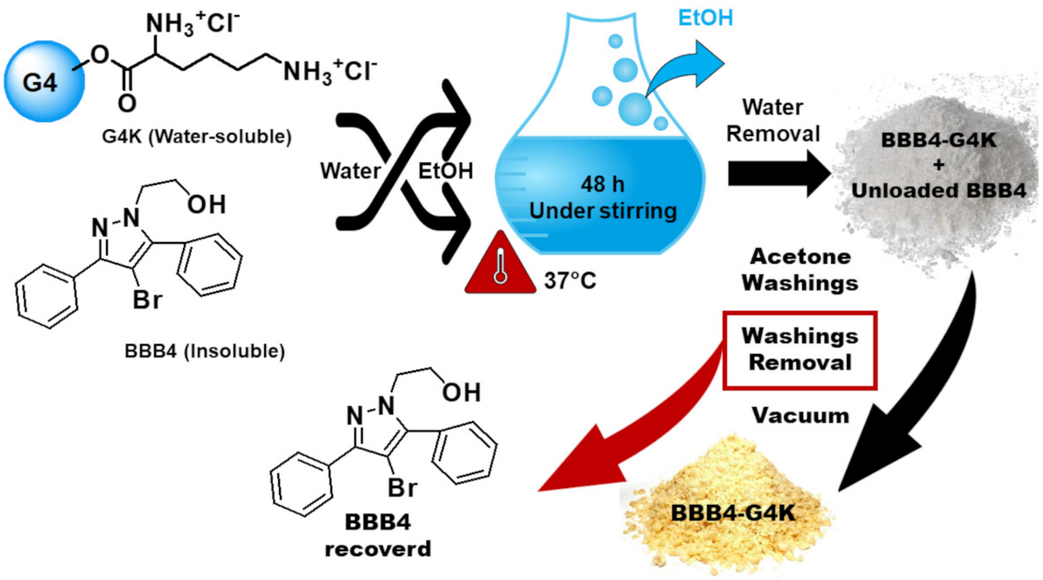

Scheme S1. Synthetic pathway to prepare BBB4-G4K NPs. G4 = fourth generation; K = lysine; BBB4 = 2-(4-bromo-3,5-diphenyl-pyrazol-1-yl)-ethanol.

The BBB4-G4K NPs were prepared according to a reported procedure modified as follows [62]. A total of 93.5 mg (6.2 μmol) of G4K dendrimer (Gen 4.0) was dissolved in 7.5 mL of deionized water (pH = 7.4). To the dendrimer water solution, a strong excess of BBB4 (98.0 mg) and a total of 6 mL of ethanol (EtOH) in three aliquots to achieve a clear solution were added. The resulting solution was incubated for 48 h at 37 °C under vigorous stirring, and following incubation, the solution was evaporated. The evaporation of the hydroalcoholic mixture was performed using a Rotavapor® R-3000 (Büchi Labortechnik, Flawil, St. Gallen, Switzerland) at 100 °C and reduced pressure. The waxy solid residue was washed under stirring for 1 h with acetone to extract the BBB4 not encapsulated. The acetone washings were separated and the BBB4-loaded dendrimer nanoparticles (BBB4-G4K NPs), purified by the free UA, were obtained as an orange, sticky solid which was stored under vacuum in a dryer (132.6 mg). Non-encapsulated BBB4 was recovered by evaporating the acetonic solution and was obtained in the form of an off-white solid (64.0 mg). The solid was recrystallized

from diethyl ether/petrol ether 1:1, and its structure was confirmed by ATR-FTIR analysis (spectrum not reported).

FTIR (ν, cm^{-1}): 3500–3000 (NH_3^+ dendrimer, OH stretching BBB4), 2985, 2880 (alkyl groups of the dendrimer and BBB4), 1736 (C=O stretching esters of the dendrimer), 1220, 1051 (C-O stretching esters of the dendrimer), 697 (C-Br stretching of BBB4).

^1H NMR (CD_3OD, 400 MHz): δ ≤ 1 (CH_3 *core* not detected), 1.00–2.00 [m, 135 H (CH_3 G1, G2, G3 and G4 of the dendrimer) + 288 H ($CH_2CH_2CH_2$ of lys)], 2.95–3.16 [m, 96H ($CH_2NH_3^+$ of lys)], 3.96–4.03 [m, 36H (CH_2 of BBB4)], 3.99 (m, 48 H, $CHNH_3^+$ of lys), 4.16–4.25 [m, 36 H, (CH_2 of BBB4)], 4.30–4.50 [m, 186 H (CH_2O of the dendrimer), 7.35–7.57 [m, 144 H (phenyl rings of BBB4)], 7.90–7.98 [m, 36 H (phenyl rings of BBB4)]. The peaks of the NH_3^+ groups of lys (288 H) and the OH groups of BBB4 (18 H) were not detectable because the protons from these groups are exchanged with the proton of CD_3OD. From ^1H NMR analysis: $C_{824}H_{1314}N_{132}O_{204}Cl_{96}Br_{18}$; MW = 21,175.8.

S1.2. Morphology of G4K and BBB4-G4K Particles

The morphology of G4K and BBB4-G4K was investigated by scanning electron microscopy (SEM) (Figure S1). In the performed experiments, samples were fixed on aluminum pin stubs and sputter-coated with a gold layer of 30 mA for 1 min, and an accelerating voltage of 20 kV was used for the sample's examination. The micrographs were recorded digitally using the DISS 5 digital image acquisition system (Point Electronic GmbH, Halle, Germany).

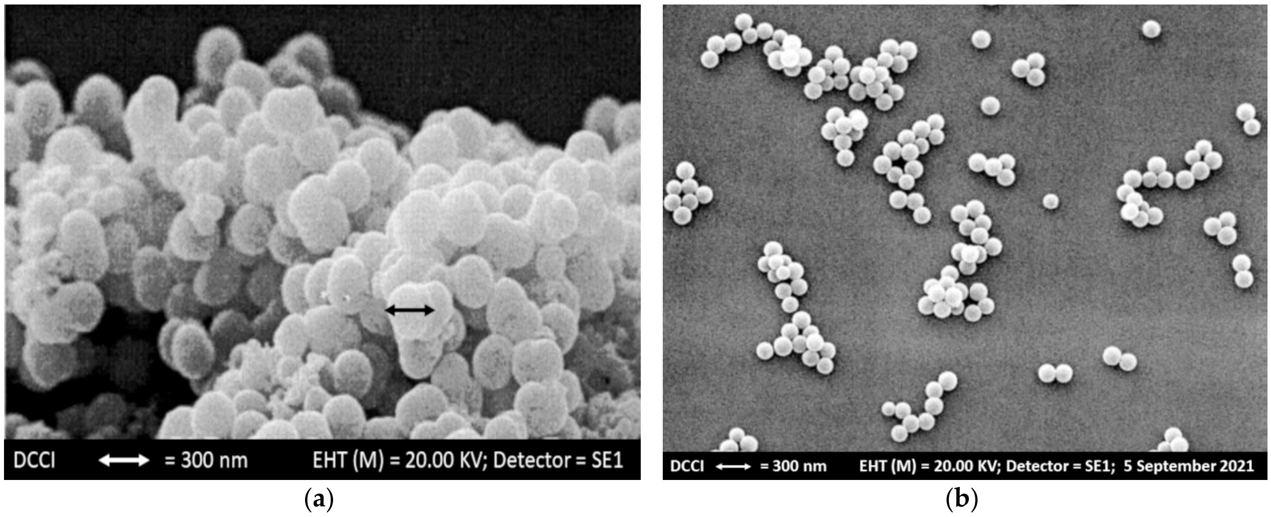

(**a**) (**b**)

Figure S1. SEM images of G4K (**a**) and BBB4-G4K (**b**) particles.

S1.3. Content of BBB4 in BBB4-G4K NPs, Drug Loading (DL%) and Encapsulation Efficiency (EE%)

S1.3.1. BBB4 Calibration Curve

An initial solution of BBB4 (1 mg/mL) was prepared in MeOH, and dilutions with MeOH were made to prepare standard solutions at concentrations of 50, 100, 200, 300, 400, and 500 μg/mL. Aliquots of 20 μL were picked up from each solution and were analysed to construct the BBB4 standard calibration curve. In particular, BBB4 in each solution was quantified using an HPLC JASCO system (Jasco Inc., Easton, MD, USA) equipped with a JASCO PU-980 pump, a JASCO UV-970-975 UV/Vis detector, and an ODS C18 column (250 × 4.6 mm, 5 μm), by detecting the absorbance (A) at λ_{abs} = 253 nm. The mobile phase consisted of a mixture of acetonitrile and 10 mM K_2HPO_4 aqueous buffer solution (15/85, *v*/*v*). The column was preconditioned for at least 20 min before the first injection. The running time was set at 20 min. The BBB4 calibration curve was obtained by a least-squares linear regression analysis of the BBB4 concentrations vs. the A signals created in the UV detector by the different concentrations of analyte (BBB4) and its equation was Eq. (S1).

$$y = 0.0369x + 0.0244 \quad \text{(S1)}$$

where y is the absorbance measured at λ_{abs} = 253 nm, and x is the BBB4 concentration (C_{BBB4}) (µg/mL).

Determinations were made in triplicate, and the A values obtained for each BBB4 concentration analysed were expressed as mean ± standard deviation (A_{mean} ± SD).

S1.3.2. Estimation of BBB4 Content in BBB4-G4K NPs

A total of 5 mg of BBB4-G4K was dissolved in 10 mL of MeOH (500 µg/mL), and the clear solution was vigorously stirred for ten minutes to promote the release of BBB4. The amount of BBB4 in the sample was quantified at λ_{abs} = 253 nm by HPLC analysis, using the same apparatus and the same conditions described in the previous section. Particularly, six aliquots (20 µL) of the solution were analysed against a blank solution of the empty dendrimer G4K. The values of DL% and EE% of BBB4-G4K were calculated from the following Equations (S2) and (S3):

$$DL\ (\%) = \frac{\text{weight of the drug in NPs}}{\text{weight of the NPs}} \times 100 \quad (S2)$$

$$EE\ (\%) = \frac{\text{weight of the drug in NPs}}{\text{inizial amount of drug}} \times 100 \quad (S3)$$

Table S1 collects the results concerning the above-mentioned determinations.

Table S1. Values of A obtained for the six aliquots and the related C_{BBB4} obtained from Eq. (S1), results concerning the concentration of BBB4 in BBB4-G4K NPs, DL%, EE%, molecular formula and MW of BBB4-G4K NPs, as well as the difference between the MW obtained by ^{1}H NMR and that computed using HPLC results, expressed as error %.

A (mAU)	C_{BBB4} (µg/mL)	BBB4 in BBB4-G4K	DL (%) EE (%)	Molecular Formula	MW	Error (%)
5.47	147.6	0.144±0.0059 mg* 38.2±1.6 mg§ 17.7±0.7#	28.8±1.2 39.0±1.6	$C_{824}H_{1314}N_{132}O_{204}Cl_{96}Br_{18}$	21072.6±240.2 [1] 21175.8 [2]	0.5 [3]
5.39	145.4					
5.50	148.4					
4.98	134.3					
5.11	137.8					
5.58	150.6					

* mg of BBB4 in 0.500 mg of BBB4-G4K NPs; § mg of BBB4 in the obtained BBB4-G4K NPs (132.6 mg); moles of BBB4 loaded per G4K mole; [1] computed using the DL% value obtained by HPLC analyses; [2] computed by the ^{1}H NMR spectrum of BBB4-G4K NPs; [3] computed on the mean.

S1.4. BBB4-G4K NPs Molecular Weight (MW)

According to a previously reported procedure [24], the MW of BBB4-G4K NPs was estimated both by its ^{1}H NMR spectrum and by the results of HPLC analyses, obtaining findings with a minimal difference (0.5%) (Table S1).

S1.5 Water Solubility of BBB4, BBB4-G4K NPs, and of the Nanotechnologically Manipulated BBB4 Released in Water

The water solubility of pristine BBB4, BBB4 in the form of BBB4-G4K NPs, and of nanoengineered BBB4 solubilized in water was determined with the shake-flask method [23]. An exactly weighted excess of BBB4 and BBB4-G4K (13.3 mg) was added with water m-Q (1.5 mL), obtaining suspensions which were maintained under vigorous stirring at room temperature, observing, for BBB4-G4K only, abundant foaming (pH = 7.4). The suspensions were stirred until producing an equilibrium between the saturated solution and undissolved BBB4 and BBB4-G4K. Then, the suspensions were centrifugated (15 min, 3500 rpm) to remove undissolved BBB4 and BBB4-G4K and drops of the supernatant solutions were observed with a Leica Galen III Professional Microscope (Taylor Scientific, St. Louis, MO, USA), without observing precipitate or differences with a drop of pure water. The solid residues were washed twice with acetone to help water removal and brought to constant weight under vacuum, obtaining, in the case of pristine BBB4, 13.24 ± 0.03 mg of insoluble material, while 3.70 ± 0.08 mg of residual was obtained in the case of BBB4-G4K NPs. The amounts of BBB4 (0.06 ± 0.03 mg) and of BBB4-G4K (9.6 ± 0.08 mg) solubilized in water were obtained for difference from the initial amount added in 1.5 mL water (13.3 mg). The experiments were made in triplicate, and the solubilities of pristine BBB4 and of BBB4 in the form of BBB4-G4K NPs were reported as mean ± SD. Additionally, the water solution obtained by dissolving BBB4-G4K was diluted to have a final volume of 10 mL with MeOH, and 20 μL aliquots were analyzed by HPLC using the same apparatus and the same conditions described in Section S1.3.1. The exact amount of BBB4 which was solubilized in water was quantified at 253 nm using the previously constructed standard calibration curve. The determinations were made in triplicate, and the BBB4 water solubility was reported as mean ± SD (Figure S2).

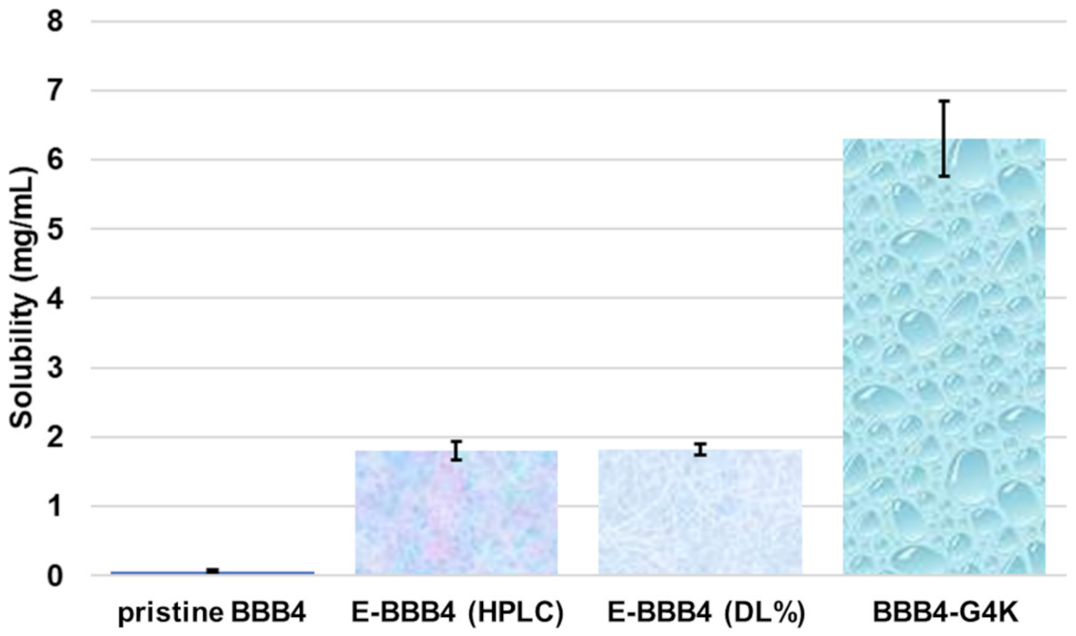

Figure S2. Water solubility of pristine BBB4, of nanotechnologically-manipulated BBB4 released in water solution [E-BBB4 (HPLC)], of BBB4 contained in BBB4-G4K NPs [E-BBB4 (DL%)], and of BBB4-G4K NPs (BBB4-G4K).

S1.6. Dynamic Light Scattering (DLS) Analysis

Particle size (in nm), polydispersity index (PDI), and zeta potential (*ζ-p*) (mV) of BBB4-G4K were measured at 25 °C at a scattering angle of 90° in m-Q water by using a Malvern Nano ZS90 light scattering apparatus (Malvern Instruments Ltd., Worcestershire, UK).

Solutions of BBB4-G4K in m-Q water were diluted to final concentrations to have 250–600 kcps. *ζ-p* value of BBB4-G4K was recorded with the same apparatus. The results from these experiments were presented as the mean of three different determinations ± SD. Concerning the particle size distribution, intensity-based results were reported

Table S2 collects the results obtained from DLS analyses on G4K and on BBB4-G4K NPs concerning their size (Z-ave, nm), polydispersity index (PDI) and Zeta potential (ζ-p).

Table S2. Results obtained from DLS analyses on G4K and BBB4-G4K NPs: particle size (Z-ave, nm), polydispersity index (PDI) and Zeta potential (ζ-p).

Physical characteristics	G4K	BBB4-G4K
Z-Ave (nm)	333.4±24.6	112.1±10.7
PDI	0.286±0.040	0.289±0.028
ζ-p (mV)	+66.1±4.7	+28.9±7.20

Z-Ave = hydrodynamic diameter; ζ-p = Zeta potential.

S.1.7. Potentiometric Titration of G4K and BBB4-G4K NPs

Potentiometric titrations were performed at room temperature to construct the titration curves of G4K and BBB4-G4K. The samples (20-30 mg) were dissolved in 30 mL of Milli-Q water (m-Q), then were treated with a standard 0.1 N NaOH aqueous solution [1.5 mL, pH = 9.34 (G4K) and 9.54 (BBB4-G4K)]. The solutions were potentiometrically titrated by adding 0.2 mL aliquots of a standard 0.1 N HCl aqueous solution, up to total 3.0 mL and measuring the corresponding pH values [63]. Titrations were made in triplicate and the determinations were reported as mean ± SD (Figure S3).

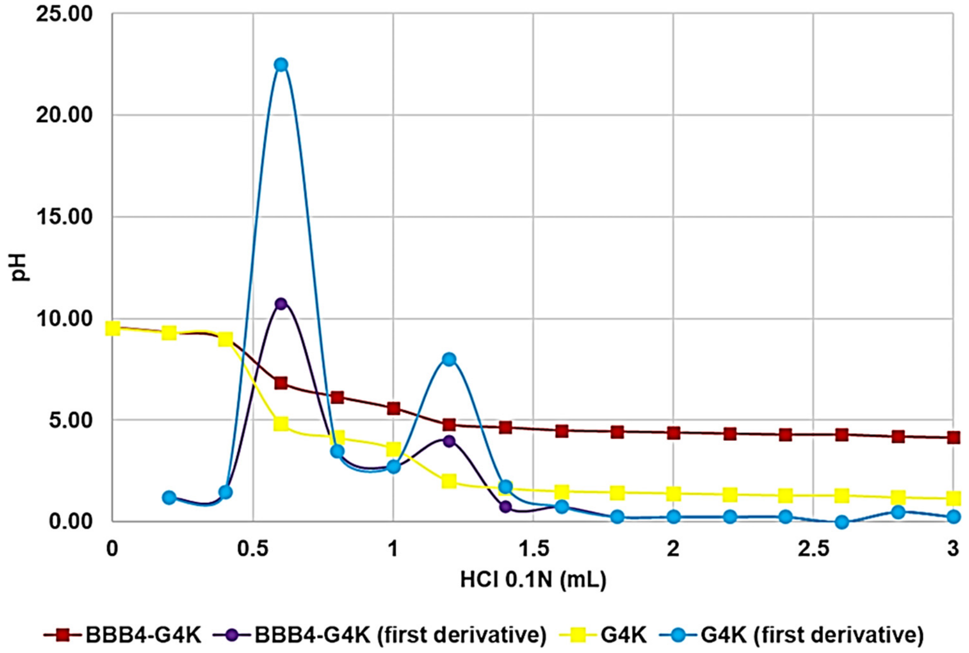

Figure S3. Titration curves (error bars not reported since difficult to detect) and first derivatives lines of the titration curves of G4K and BBB4-G4K.

S1.8. In Vitro BBB4 Release Profile from BBB4-G4K NPs

In vitro release of BBB4 from BBB4-G4K NPs was investigated using the dialysis bag diffusion method. An exactly weighted amount of BBB4-G4K (10 mg) was dissolved in 2 mL of 0.1 M phosphate-buffered saline (PBS, pH = 7.4), which should assure the dissolution of the complex. The solution was then placed into a pre-swelled T2 tubular cellulose dialysis bag (flat width = 10 mm, wall thickness = 28 μm, V/cm = 0.32 mL) with a nominal molecular weight cut-off (MWCO) of 6000-8000 Da (Membrane Filtration Products, Inc., Seguin, TX, USA) and dipped into 20 mL of 0.1 M PBS, pH 7.4, at 37 °C with gentle stirring for 24 h. At predetermined time intervals (1 h, 2 h, 3 h, 4 h, 5 h, 6 h, 8 h, 10 h, 12 h, 24 h), 1 mL was withdrawn from the incubation medium and was analyzed by HPLC using the same apparatus and the same conditions described in Section 2.7.1 to determine the BBB4 concentration. The exact amount of BBB4 present in the samples was quantified at 253 nm, and the results were reported as the mean ± SD of three determinations. After sampling, an equal volume of fresh PBS was immediately replaced into the incubation medium.

The concentration of BBB4 released from BBB4-G4K NPs was expressed as a cumulative release percentage (%) of the total amount of BBB4 present in the BBB4-G4K NPs (according to the DL% value). The percentage BBB4 cumulative releases (CR %) were plotted as a function of time obtaining the curve of BBB4 release profile (Figure S4).

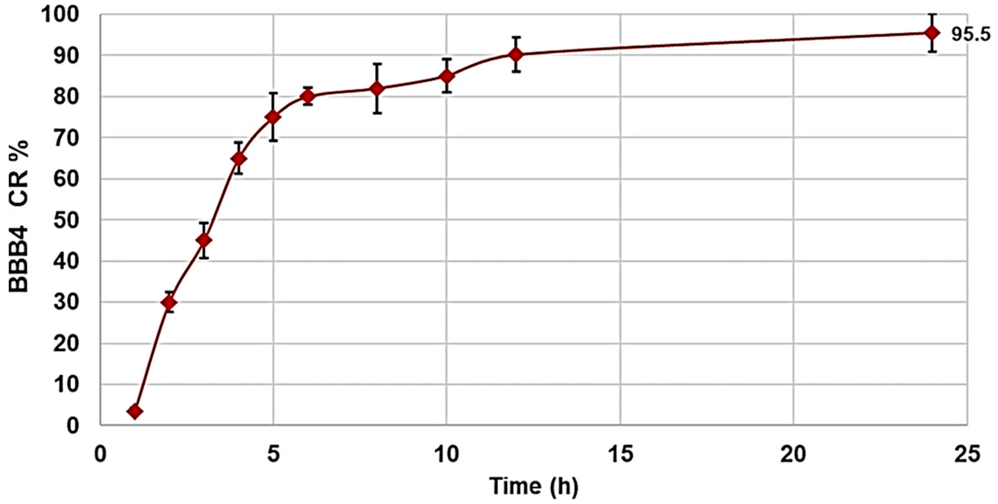

Figure S4. Percentage BBB4 cumulative release (CR %) at pH 7.4 monitored for 24 hours.

The kinetics and the main mechanisms which govern the release of BBB4 from BBB4-G4K NPs were determined by fitting the CR% curve data with the equations of zero order model (% cumulative drug release vs time), first-order model (log % cumulative drug remaining vs time), Hixson-Crowell model (cube root of % cumulative drug remaining vs time), Higuchi model (% cumulative drug release vs square root of time), and Korsmeyer-Peppas model (Ln % cumulative drug release vs Ln of time). The highest value of the coefficient of determination (R^2) of the equations of linear mathematical models, was considered as the parameter to determine which model better fits the release data. R^2 values were 0.5208 (zero order), 0.8603 (first order), 0.6933 (Korsmeyer-peppas model), 0.7246 (Hixson Crowell model) and 0.7232 (Higuchi model) thus establishing that the BBB4 release best fitted with first order kinetic model (Figure S5).

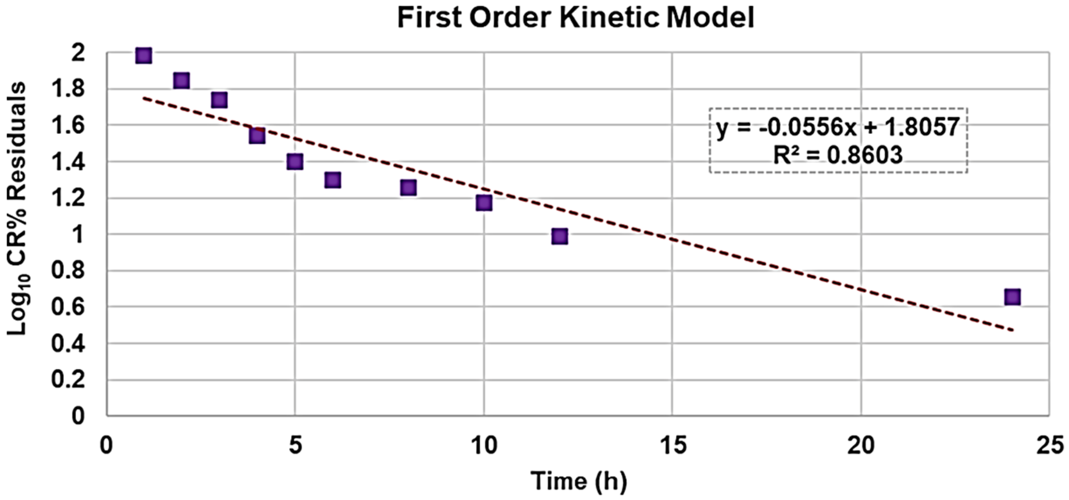

Figure S5. Linear regressions of the First Order kinetic mathematical model with the related equation and R^2 value.

Section S2. Biological Investigations.

S2.1. Cytotoxicity Studies Performed for G4K on HeLa Cells

Dose-dependent studies of cytotoxicity were performed for the reservoir dendrimer G4K. The cytotoxicity of G4K was evaluated in vitro on HeLa cell lines purchased by Termofischer Scientific (Rodano, Milan, Italy). Briefly HeLa cells were increased in Dulbecco's Modified Eagle Medium (DMEM) enriched with Fetal Bovine Serum (FBS, 10%), non-essential amino acids (1%) and antibiotics (1%, penicillin and streptomycin) and maintained in an atmosphere containing 5% CO_2 at 37 °C. The cells were seeded at the density of 2 × 104 cells per well in a 24-well plate and in 4-wells slides in 500 μL of medium and incubated at 37 °C for 72 h. Subsequently, the cells were incubated with increasing concentrations (5–40 μM) of G4K at 37 °C for 24 h. Then 10 μL MTT [3-(4,5-dimethylthiazol-2-yl)-2,5-diphenyl-2H-tetrazolium bromide] was added into each well and after 4 h, the medium and MTT were discarded and 100 μL dimethyl sulfoxide (DMSO) was added into each well. Finally, optical density at 490 nm was measured on a Termofischer Scientific microplate reader (Rodano, Milan, Italy) to determine cells viability (%). Paclitaxel was essayed in the same condition as a positive control. Determinations were made in triplicate and results were expressed as mean percentage of the control (untreated cells) ± standard deviation (SD) (Figure S6).

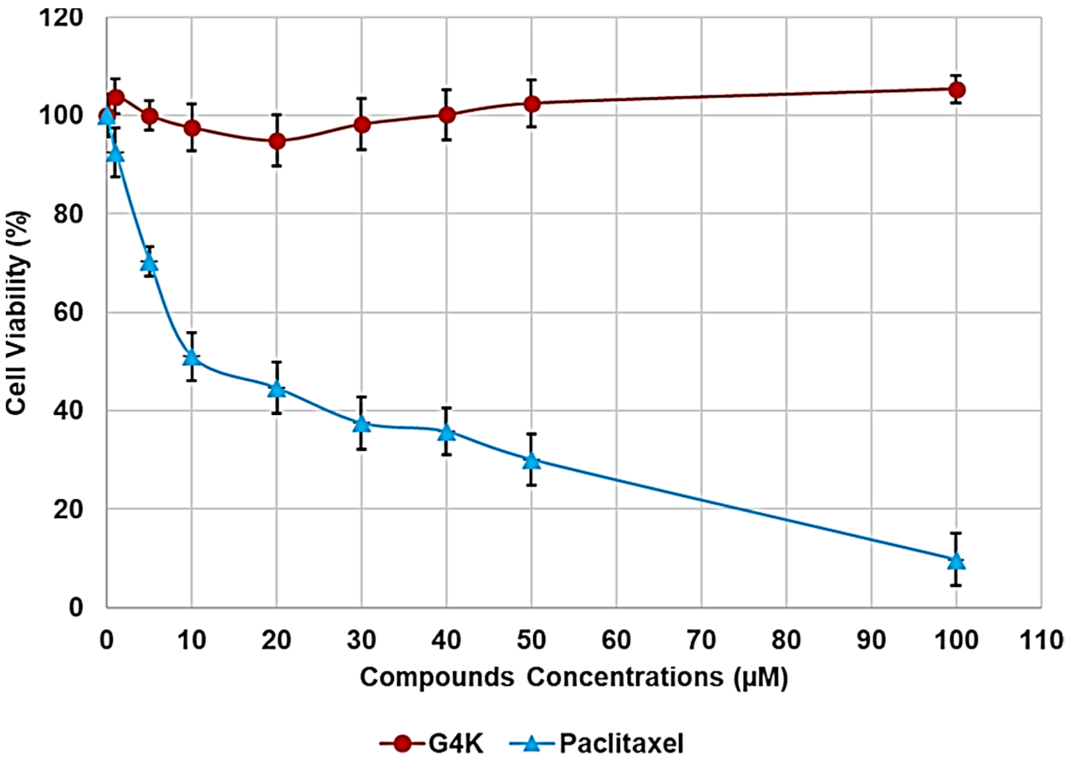

Figure S6. Cells viability of HeLa cells exposed for 24 h to G4K and Paclitaxel at concentration 0-100 μM.

S2.2. Cytotoxicity Studies Performed for G4K, BBB4 and BBB4-G4K NPs on HaCaT Cells

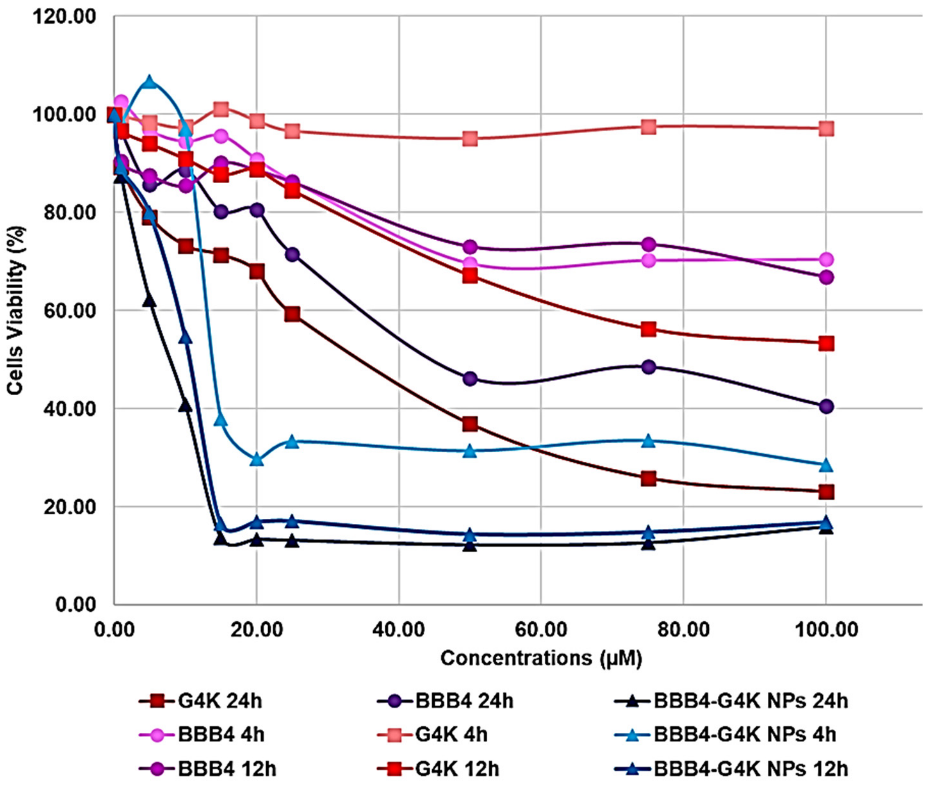

Figure S7. Curves of the cell's viability (%) as a function of the compound's concentrations for all samples and for all times of exposure.
